# Learning: Not Just the Facts, Ma'am, but the Counterfactuals as Well

**DOI:** 10.1371/journal.pbio.1001092

**Published:** 2011-06-28

**Authors:** Michael L. Platt, Ben Hayden

**Affiliations:** Department of Neurobiology, Duke University Medical Center, Durham, North Carolina, United States of America

Our brains allow us to consider rewards and other scenarios that could have happened but did not. Such counterfactual outcomes can influence our choices and hasten learning. A series of recent studies has begun to untangle the neural circuitry responsible for monitoring counterfactual outcomes. Here, we summarize several recent complementary discoveries, including a new article in the current issue of *PLoS Biology*. Neurons in several brain areas that process directly experienced rewards respond to counterfactual information about rewards as well. Among these brain regions, the frontal pole appears to be most specialized, and carries a decision variable representing the value of the best alternative option. Together, these findings suggest that counterfactual learning and thinking build upon scaffolding circuits that evolved to learn from direct experience.

In the hit 1950s television series *Dragnet*, Detective Joe Friday methodically solved crimes by slowly accumulating knowledge of what really happened, famously stating, “All we want are the facts, ma'am.” In the last two decades, neuroscience has revealed some of the mechanisms that allow us to go beyond Joe Friday's trademark phrase, to reflect on our experiences and imagine different possibilities, and, with this understanding as a foundation, has begun to peek at the circuitry that lets us understand what might have been [1]–[6].

Reinforcement learning (RL) models posit that decision-makers carry internal representations of reward states in the world, and update these representations based solely on direct experience of the outcome of their actions—just the facts. A chief appeal of RL is that it can explain so much of behavior using such a limited palette—expectations, actions, and outcomes. Much of the behavior and decision-making observed in animals can be explained using only RL.

For humans, however, choices clearly depend on more than just our own direct experience. We have sudden insights, we selectively ignore information we don't like, we have a plethora of biases, and we can even take into account rewards that we could have gotten had things turned out differently or had we made different choices. Thinking about such alternative outcomes is often known as counterfactual, fictive, or hypothetical reasoning.

Understanding the neurobiology of counterfactual reasoning provokes natural philosophical interest [7]–[9], but has practical importance as well; impaired counterfactual thinking is a marker for addiction, depression, and obsessive-compulsive disorder. Indeed, retraining patients' counterfactual thought patterns can improve behavioral function in these diseases [6],.

So, onto the trim RL framework, we can now add counterfactual outcomes, which can contribute to learning and decision-making in ways formally analogous to direct experience [11]. Counterfactual thinking has recently become a major focus of neuroscientific study.

One of the first studies to examine this question used fMRI to scan participants' brains while they played a simulated stock market task [1]. Participants chose how much to wager, made a choice, and then found out how much the market had changed, revealing both how much they did win and how much they could have won or lost had they wagered more or less. The difference between how much participants won and how much they could have won, or fictive error, strongly activated the ventral caudate nucleus. This group subsequently found that the translation of fictive reward information into behavior is compromised in addition. Chiu and colleagues found that fictive errors activate the caudate in smokers as well but that these signals do not influence subsequent choices [6], implicating impaired fictive learning in real-life problems like addiction and gambling (and playing the stock market as well).

The appeal of RL derives both from its power and its generality—it drives behavior in animals as diverse as slugs and stock traders. But, while humans clearly and readily imagine counterfactual outcomes, until recently there was no experimental evidence that animals did so as well, thus raising the possibility that fictive thinking is uniquely human and thus reliant on uniquely human brain mechanisms. To address this question, we devised a novel task capable of revealing whether rhesus macaques recognize and respond to fictive outcomes. On each trial, monkeys chose one of eight possible targets, and then, before the reward was given, saw a display indicating the reward each target would have yielded if it had been chosen. We then examined neural responses in dorsal anterior cingulate cortex (dACC), a brain area implicated in learning. We found that many individual neurons responded to both real and fictive information about rewards, and did so almost exclusively using the same coding scheme for both types of outcomes.

Our results suggested that dACC carries a conjoint representation that is agnostic to reward type, and raise the question of how and where real and fictive reward information are combined into an abstract reward signal such as the one observed in dACC. Two candidate structures for this are input structures to dACC: the orbitofrontal cortex (OFC) and dorsolateral prefrontal cortex (dlPFC). In a parallel study, Abe and Lee examined neural activity in these two structures in a task that pitted macaques against a computer in a weighted rock-paper-scissors game [4]. In this three-option choice task, monkeys adjusted their behavior in response to rewards they could have received had they chosen differently [12], consistent with fictive learning. Abe and Lee found that neurons in both OFC and dlPFC code unchosen rewards. They found a strong statistical interaction between fictive outcome and saccade direction in dlPFC but not in OFC (nor did we observe one in dACC), suggesting that dlPFC may serve as a locus for transforming information about hypothetical rewards into specific actions.

These studies suggest that specific brain areas represent fictive outcomes, and thus pave the way for asking bigger questions about counterfactual learning. For example, why does the brain monitor counterfactual outcomes? How are these outcomes distinguished from real outcomes? And how are they integrated into subsequent decisions? A new study by Boorman and colleagues in the current issue of *PLoS Biology* directly addresses these questions [13]. The authors used fMRI to measure brain activity in humans performing a three-option gambling task. Each option was associated with a specific magnitude of reward and a probability of getting that reward. Following each choice, they were told whether each option—the one chosen and the two unchosen—would have paid out.

Boorman and colleagues focused on the lateral frontopolar cortex (lFPC). Earlier studies indicate that lFPC tracks values of alternative courses of action [14],[15]. Extending these earlier results, the authors report that lFPC tracks outcomes of unchosen options in this three-option gambling task.

Such outcomes are obviously counterfactual. Indeed, the outcome signals are counterfactual reward prediction errors—signals that are likely to drive counterfactual learning, just as reward prediction errors drive reinforcement learning. However, the signals in lFPC are more complex than this, and more interesting. To solve the task, subjects must monitor rewards obtained from each option and accumulate this information over multiple trials—so that they can estimate the probability of each target. The BOLD signal in lFPC reflects not just the most recent trial, but the accumulated estimate of the value of the second-best option. These counterfactuals thus form what appears to be a decision variable reflecting the need to adjust to a new strategy [16]. Thus, for example, when the value of the best option rises, the BOLD in lFPC falls, since this option is less favored, relative to the top option. Even more intriguingly, when the value of the third option falls, the BOLD in lFPC rises, since now the second option is relatively more valuable. This finding suggests that lFPC is not solely interested in comparing the best two options, but instead represents the value of the second option, in the broader context of the options available in the environment.

These results may have importance for understanding both depression and obsessive-compulsive disorder. Both diseases, which are highly comorbid, are associated with an inability to switch from maladaptive behavioral patterns to more adaptive ones (e.g., [17]). It is possible that these diseases derive from difficulties adjusting to the second best option or in monitoring it. Given the broad changes to frontal lobe function associated with these diseases, the study of fictive learning provides a possible entrée into understanding and potentially treating depression and obsessive-compulsive disorder.

Boorman and colleagues also found similar results in two other brain regions, the dorsomedial frontal cortex and the posteromedial cortex. These findings suggest that these three regions comprise a network for monitoring the value of unchosen options, and raise the natural question of whether these regions have distinct roles in fictive learning. Responses in these three regions stand in marked contrast to the ventromedial prefrontal cortex, which carries information about the chosen option [14]. These results also contrast with results obtained in studies of regret processing in the OFC and striatum that found that activity is correlated with the difference between the obtained and unobtained outcomes. Thus, the study by Boorman and colleagues suggests that lFPC, premotor cortex, and dorso-medial prefrontal cortex do not mediate regret per se, but instead contribute to the use of counterfactual information to guide changes in behavior.

What's next? For one thing, fictive reward processing is a thorny topic—unobtained rewards can be unobtained either because they were chosen but not received or because they were not chosen. These different types of fictive outcomes may have distinct neural substrates. Certainly, the emotions associated with them are distinct: psychologists use the terms disappointment for chosen but unobtained rewards and regret for unchosen but unobtained rewards. Relatedly, it will be necessary to identify the linkage between unobtained rewards and the emotions they evoke. From a comparative evolutionary point of view, the extent to which different animals monitor fictive rewards needs to be characterized, and learning models updated to reflect this information. Indeed, it will be important to link these ideas with model-based reinforcement learning as well [18]. Finally, it will be necessary to continue to use our emerging understanding of the neurobiology of fictive learning to treat the very real diseases that bedevil so many people.

## Abbreviations

dACC: dorsal anterior cingulate cortex
dlPFC: dorsolateral prefrontal cortex
lFPC: lateral frontopolar cortex
OFC: orbitofrontal cortex
RL: reinforcement learning
